# Patient-perceived and practitioner-perceived barriers to accessing foot care services for people with diabetes mellitus: a systematic literature review

**DOI:** 10.1186/s13047-022-00597-6

**Published:** 2022-12-16

**Authors:** Megan McPherson, Matthew Carroll, Sarah Stewart

**Affiliations:** 1 Department of Podiatry, Faculty of Health and Environmental Sciences, Auckland University of Technology, 90 Akoranga Drive, Northcote, Auckland, New Zealand; 2 Active Living and Rehabilitation: Aotearoa New Zealand, Health and Rehabilitation Research Institute, School of Clinical Sciences, Auckland University of Technology, Auckland, New Zealand

**Keywords:** Diabetes, Foot care, Podiatry, Service access, Barriers

## Abstract

**Background:**

Foot-related complications are common in people with diabetes mellitus, however foot care services are underutilized by this population. This research aimed to systematically review the literature to identify patient and practitioner-perceived barriers to accessing foot care services for people with diabetes.

**Methods:**

PRISMA guidelines were used to inform the data collection and extraction methods. CINAHL, MEDLINE, and Scopus databases were searched in March 2022 to identify original research articles that reported on barriers to accessing diabetes foot care services from the patient and/or practitioner perspective. Both quantitative and qualitative studies were included. The methodological quality of included studies was assessed using the Critical Appraisal Skills Program (CASP) tool for qualitative/mixed methods studies or the National Heart, Lung & Blood Institute (NHLBI) tool for quantitative studies. Following data extraction, content analysis was used to identify reported barriers. Themes and subthemes were presented separately for patient-perspectives and practitioner-perspectives. A narrative summary was used to synthesize the findings from the included studies.

**Results:**

A total of 20 studies were included. The majority of CASP and NHLBI criteria were met by most studies, indicating good overall methodological quality. Three predominant themes emerged from the patient perspective that represented barriers to accessing foot care services: lack of understanding, socioeconomic factors, and lack of service availability. Four themes emerged from the practitioner perspective: poor interprofessional communication, lack of resources, lack of practitioner knowledge, and perceived patient factors.

**Conclusions:**

This study has identified a number of barriers to accessing foot care services from both the patient and practitioner perspectives. Although patients focused predominantly on patient-level factors, while practitioners focused on barriers related to the health care system, there was some overlap between them. This emphasizes the importance of recognising both perspectives for the future integration of policy changes and access facilitators that may help to overcome these barriers.

## Background

Foot-related complications, including ulceration, infection, and amputation are common in people with diabetes [1]. The five-year post-amputation mortality rate in people with diabetes is 57% (increasing to 70% in those with associated comorbidities) [1, 2]. Many diabetes-related foot complications, including amputation, are preventable with early management and timely access to diabetes foot care services [2]. Current guidelines emphasize the role of the health care provider in identifying, educating, and managing risk factors of diabetic foot disease [3]. However, foot care services for people with diabetes are underutilized, with research suggesting these services are more readily accessed post-amputation [4].

Access to health care is a complex concept determined by adequate supply and availability of services as well as whether the services are affordable, physically accessible, and socially and culturally acceptable [5]. Barriers to accessing health care services can occur at both the patient-level (when the person with diabetes needs to access health services), at a health system-level (involving the responsibility of health professionals to make key decisions and appropriate specialist referrals), or within the structure of the health system itself [6, 7]. These barriers may be identified through both patient- and practitioner-perspectives, which are important in enabling discussion, implementation, and change to improve access to foot care services for this population. The aim of this study was to systematically review the literature to identify patient and practitioner-perceived barriers to accessing foot care services for people with diabetes.

## Methods

A systematic search following the Preferred Reporting Items for Systematic Reviews and MetaAnalyses (PRISMA) statement guidelines was conducted [8]. For the purpose of this review, the phrase ‘foot care service’ included those provided by podiatrists, diabetes nurses, general practitioners, or any other health practitioners managing patients with diabetes.

### Search strategy

An electronic database search was conducted in March 2022 of CINAHL, MEDLINE, and Scopus databases using the following terms: (diabet*) AND (podiatr*, OR foot, OR “foot care”) AND (barrier*). These databases were selected to ensure an extensive search across a range of journal articles in biomedicine, health, nursing, and allied health. One reviewer (MM) conducted the literature search and exported all retrieved studies into Rayyan (http://rayyan.qcri.org), an online literature review application. After removal of duplicates, titles and abstracts of included studies were screened independently by two reviewers (MM, SS). Studies were eligible for inclusion if they: reported on barriers to accessing diabetes foot care services; included participants with diabetes or practitioners providing foot care services to people with diabetes (in order to provide patient perspectives and practitioner perspectives, respectively); and were full-text original qualitative or quantitative studies that were published in English. Studies were excluded if they did not report on barriers to access of diabetes foot care services. Studies reporting on barriers to self-management, general diabetes care, were not published in English, were conference papers, or did not report original research were also excluded. The full texts of the studies deemed eligible from title and abstract screening were then screened again by the two independent reviewers (MM, SS) against the above criteria to confirm eligibility. Any disagreements were resolved by discussion.

### Quality assessment

Quality assessment tools were used to assess the methodological quality of the studies included. Quantitative studies were assessed using the National Heart, Lung, and Blood Institute (NHLBI) quality assessment tool for Observational Cohort and Cross-sectional Studies [9]. This 14-item tool includes items related to internal validity that allow evaluation of potential bias or flaws in the methods and implementation of each study. Items were scored as ‘yes’ if satisfied, ‘no’ if not satisfied and ‘can’t tell’ if it was not clear whether the item was satisfied or not. Qualitative studies were assessed using the Critical Appraisal Skills Programme (CASP) Qualitative Studies Checklist [10]. This 10-item checklist was developed to evaluate validity, results and clinical relevance of each study. Items were scored as ‘yes’ if the criterion was met, ‘no’ if not met, ‘NR’ if not reported, and ‘NA’ if not applicable. Quality assessment was performed independently by two reviewers (MM, SS) and disagreements were resolved by discussion.

### Data extraction

Following a reliability exercise between two reviewers (MM, SS) to ensure consistency with data extraction, data from all included studies were extracted by a single reviewer (MM) into a standardized Microsoft Excel spreadsheet, including study characteristics (first author name, year of publication, country, study design, and data collection methods), and participant characteristics (sample size, description of patient and/or practitioner cohorts). Data relating to barriers to accessing foot care services was extracted separately for patient and practitioner perspectives in the form of verbatim participant quotes and author summaries of participants’ experiences (for qualitative research) or descriptive statistics (for quantitative research).

### Data synthesis

A content analysis approach was used to identify reported barriers and analyze the presence, meanings and relationships of key concepts [11]. Following familiarization with the data, the researcher (MM) identified meaning units within the extracted data which were labeled with relevant codes using an inductive approach. Extended meaning units were then condensed before key themes were identified related to distinct subthemes. Themes and subthemes were presented separately for patient-perspectives and practitioner-perspectives. Illustrative quotes from included studies were also selected to provide evidence for each theme. In addition, a quantitative approach to content analysis was also undertaken to tabulate and count frequencies of the identified themes (barriers). A theme cloud was used to provide a visual summary of the frequencies of each subtheme as they appeared across the included studies and how the subthemes interconnected. To enhance validity, researcher triangulation was undertaken between two researchers (MM, SS) throughout the analytic process, including regular discussions regarding coding, identification of themes and subthemes, and interpretation of meaning.

## Results

### Study characteristics

**Fig. 1:**
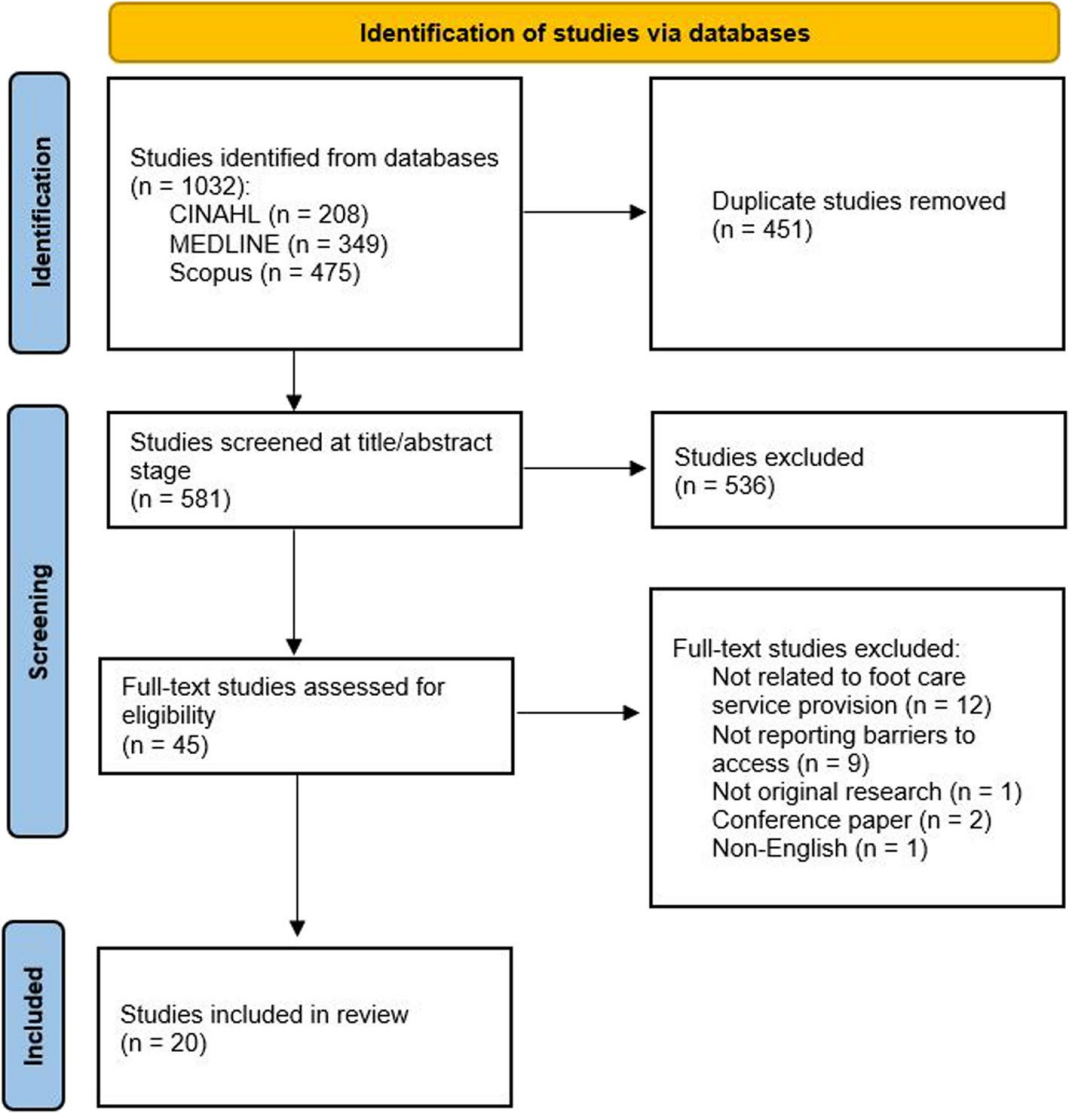
PRISMA Flow Chart

The literature search identified 1032 articles. The number of studies included and excluded at each stage of the review process are presented in Fig. 1. A total of 20 studies were found to be eligible for inclusion. Characteristics of the included studies are presented in Table 1. The included studies were conducted in ten countries: United States of America (*n* = 6), Australia (*n* = 5), United Kingdom (*n* = 2), Jordan (*n* = 1), United Arab Emirates (*n* = 1), Ireland (*n* = 1), Barbados (*n* = 1), India (*n* = 1), China (*n* = 1), and Italy (*n* = 1). The majority of studies included participants from tertiary (*n* = 6) or primary (*n* = 6) settings. Two studies included participants from secondary care settings, and the remainder included participants from a mixture of primary, secondary and/or tertiary care. Thirteen studies discussed patient-perceived barriers and ten studies discussed practitioner-perceived barriers. Three studies included both perspectives. The sample sizes ranged from 7 to 11,274 participants. All patient participants had diabetes, and many had previously experienced or were being treated for foot ulceration or lower limb amputation. Practitioner participants included podiatrists, nurses, general practitioners, diabetes specialists, and diabetes educators. Studies used surveys, questionnaires, unstructured and semi-structured interviews, focus groups, and workshops to collect data. Four studies used quantitative methodology, two used mixed-methods, and the remaining 14 studies used a qualitative approach.

**Table 1:** Study and participant characteristics

Study	Location	Setting	Participant characteristics		Study design and methodology
			Patients	Practitioners	
Abu-Qamar et al., 2011 [12]	Jordan	Secondary	*N* = 7 (4 males, 3 females) Patients with diabetes receiving treatment from a foot burn injury in secondary healthcare facilities	NA	Qualitative Unstructured interviews
Alhyas et al., 2013 [13]	UAE (Abu-Dhabi)	Tertiary	NA	*N* = 9 (4 males, 5 females) Practitioners working in a tertiary hospital diabetes center (3 diabetes specialists, 2 diabetes educators, 2 nurses, 1 dietician, and 1 podiatrist)	Qualitative Semi-structured interviews
Bergin et al., 2009 [14]	Australia (Victoria)	Primary	NA	*N* = 69 Podiatrists from community-based podiatry departments in community health centres	Quantitative Self-administered Footcare Provider Survey
Burden, 1999 [15]	England (Leicester)	Primary & secondary	NA	*N* = 15 7 nurses, 2 chiropodists, 1 orthotist, 2 diabetologists, 2 GPs, and 1 public health consultant	Qualitative Semi-structured questionnaire
Cuestra-Briand et al., 2014 [16]	Australia (Perth)	Primary	*N* = 38 (10 male, 28 female; 20 non-indigenous, 18 indigenous) People with diabetes living in suburbs of socioeconomic disadvantage	NA	Qualitative Focus groups, semi-structured interviews
Delea et al., 2015 [17]	Ireland (Republic of)	Tertiary	*N* = 10 (all male) Patients with diabetes and active foot disease or lower limb amputation	NA	Qualitative Semi-structured interviews
Devlin et al., 2003 [18]	Australia	Primary, tertiary, & secondary	*N* = NR People with diabetes with a history of lower limb ulceration or amputation (interviews)	*N* = NR Key stakeholders and healthcare workers (diabetes educators, podiatrists, GPs, an endocrinologist, a general surgeon, a vascular surgeon, a community care manager, a project officer and pharmacists)	Mixed-methods Surveys, workshops, interviews
Fayfman et al., 2020 [19]	USA (Atlanta, Georgia)	Tertiary	*N* = 40 (35 male, 5 female) Patients with diabetes, with current or prior foot ulceration and/or minor amputations (below ankle) who were at high-risk for re-ulceration and further limb loss	NA	Mixed methods Quantitative survey, focus groups
Flattau et al., 2021 [20]	USA (Bronx, New York)	Primary and tertiary	*N* = 16 (8 males, 8 females) People with current or recent diabetic foot ulcers, predominantly from racial and ethnic minority groups	NA	Qualitative Semi-structured interviews
Guell et al., 2015 [21]	Barbados	Primary	*N* = 9 (6 males, 3 females) Patients with diabetes	*N* = 11 4 doctors, 4 nurses and 2 podiatrists, and 1 private GP	Qualitative, exploratory Semi-structured interviews
Harrison-Blount et al., 2014 [22]	India	Tertiary	NA	*N* = 11 Doctors or healthcare professionals in positions of heads or assistant heads of a department, and regularly involved in foot health problems	Qualitative Focus groups, observations, individual conversations
Littman et al., 2021 [23]	USA	Tertiary	*N* = 61 (all males) Veterans with diabetes who had undergone a toe amputation	NA	Qualitative Semi-structured interviews
Liu et al., 2021 [24]	China (Beijing)	Tertiary	*N* = 41 (12 males, 29 females) Patients with diabetes, receiving treatment for diabetes foot complications at a tertiary hospital	NA	Qualitative Semi-structured interviews
Meloni et al., 2021 [25]	Italy	Secondary	NA	*N* = 99 Italian diabetes centres dedicated to diabetes foot care	Quantitative Survey
Mirmiran et al., 2000 [26]	USA (San Francisco Bay Area)	Primary & secondary	*N* = 392 (179 males, 213 females) Patients with diabetes who are members of the American Diabetes Association	NA	Quantitative Survey
Mullan et al., 2021 [27]	Australia	Primary	NA	*N* = 16 Primary healthcare professionals (2 GPs and 14 diabetic educators)	Qualitative Semi-structured interviews
Pankhurst et al., 2018 [6]	United Kingdom	Primary & secondary	NA	*N* = 425 Healthcare professionals attending the 2015 and 2016 Masterclass multidisciplinary diabetes foot conferences at King’s College Hospital	Qualitative Participants were asked to write down, in free text, the issues which they considered to constitute barriers to diabetic foot care
Parikh et al., 2013 [28]	USA	Primary	*N* = 11,274 (5490 males, 5784 females) Adults that reported a diagnosis of diabetes and coronary heart disease	NA	Quantitative 2007 Centers for Disease Control Behavioral Risk Factor Surveillance Survey
Sutherland et al., 2020 [29]	USA	Primary & secondary	*N* = 5 Patients/caregivers with diabetic foot ulcers	*N* = 39 6 rural primary care providers, 12 rural specialists, 12 urban specialists, 9 support staff	Qualitative Semi-structured interviews
Wong et al., 2005 [30]	Australia (Torres Straight and Northern Peninsula Area of Far North Queensland)	Primary	*N* = 67 (26 males, 41 females) Torres Straight Islanders with diabetes	NA	Qualitative Focus groups, interviews

*NA* not applicable, *NR* not reported, *GP* general practitioner

### Quality assessment

**Fig. 2:**
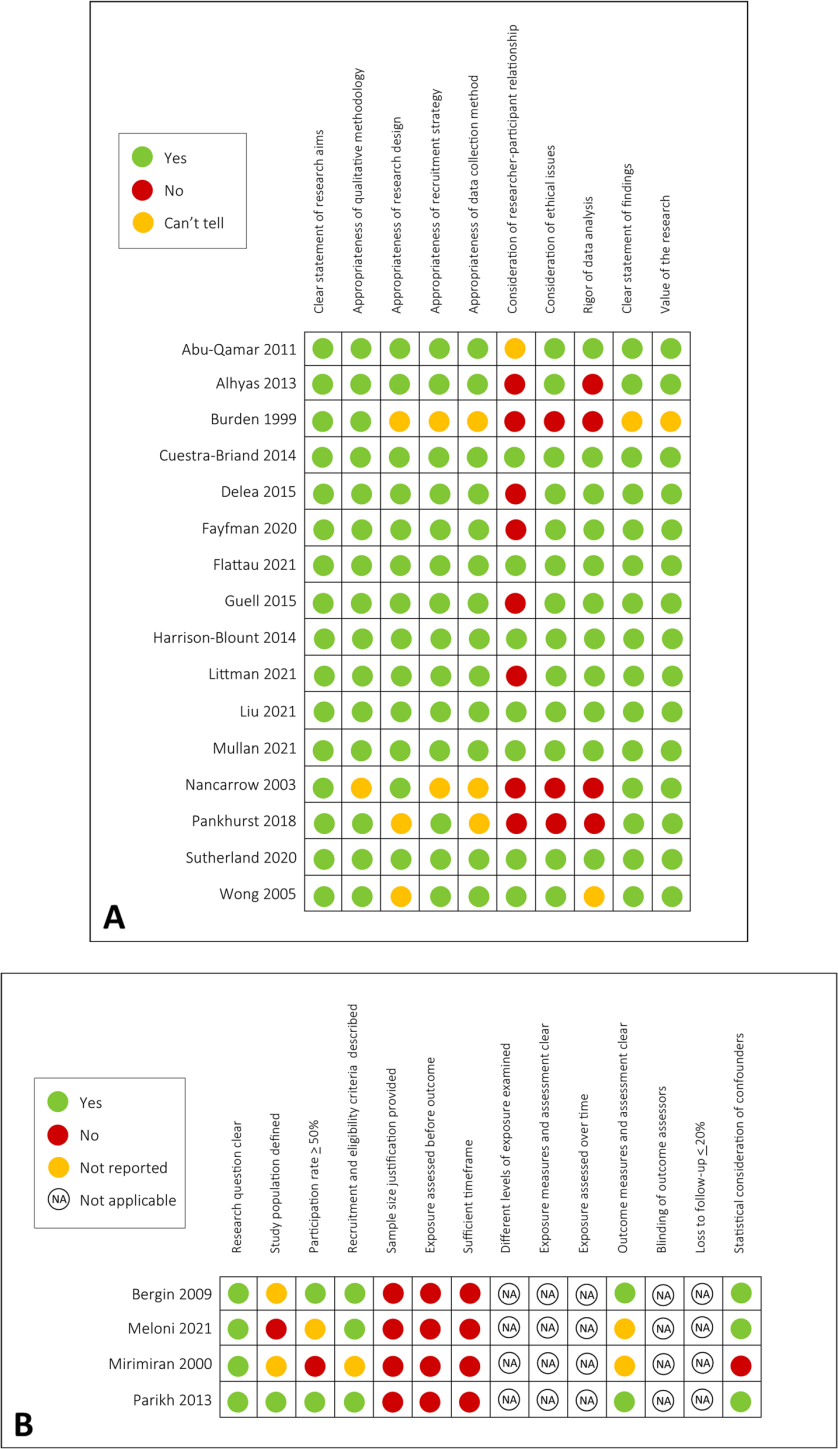
Results of the quality assessment. **A** Qualitative and mixed methods studies assessed using the CASP tool. **B** Quantitative studies assessed using the NHLBI tool

The CASP tool was used to evaluate 16 qualitative and mixed methods studies (Fig. 2a), and the NHLBI tool was used to evaluate four quantitative studies (Fig. 2b). Among the qualitative studies, the methodology, research design, recruitment strategy, data collection methods, and reporting and relevance of findings were of high quality across most studies. However, half of the qualitative studies did not provide information about the researcher-participant relationship, and only three provided consideration of ethical issues, with four using a rigorous data analysis method. Among the four quantitative studies, the majority provided a clear research question and recruitment criteria. Only two studies provided a clear description of their outcome measures assessment, and no studies provided a sample size justification. As all four studies were cross-sectional studies, five items (related to the assessment of exposure measures, assessor blinding, and loss to follow up) on the NHLBI quality assessment tool were not applicable to these studies.

### Study findings

**Fig. 3:**
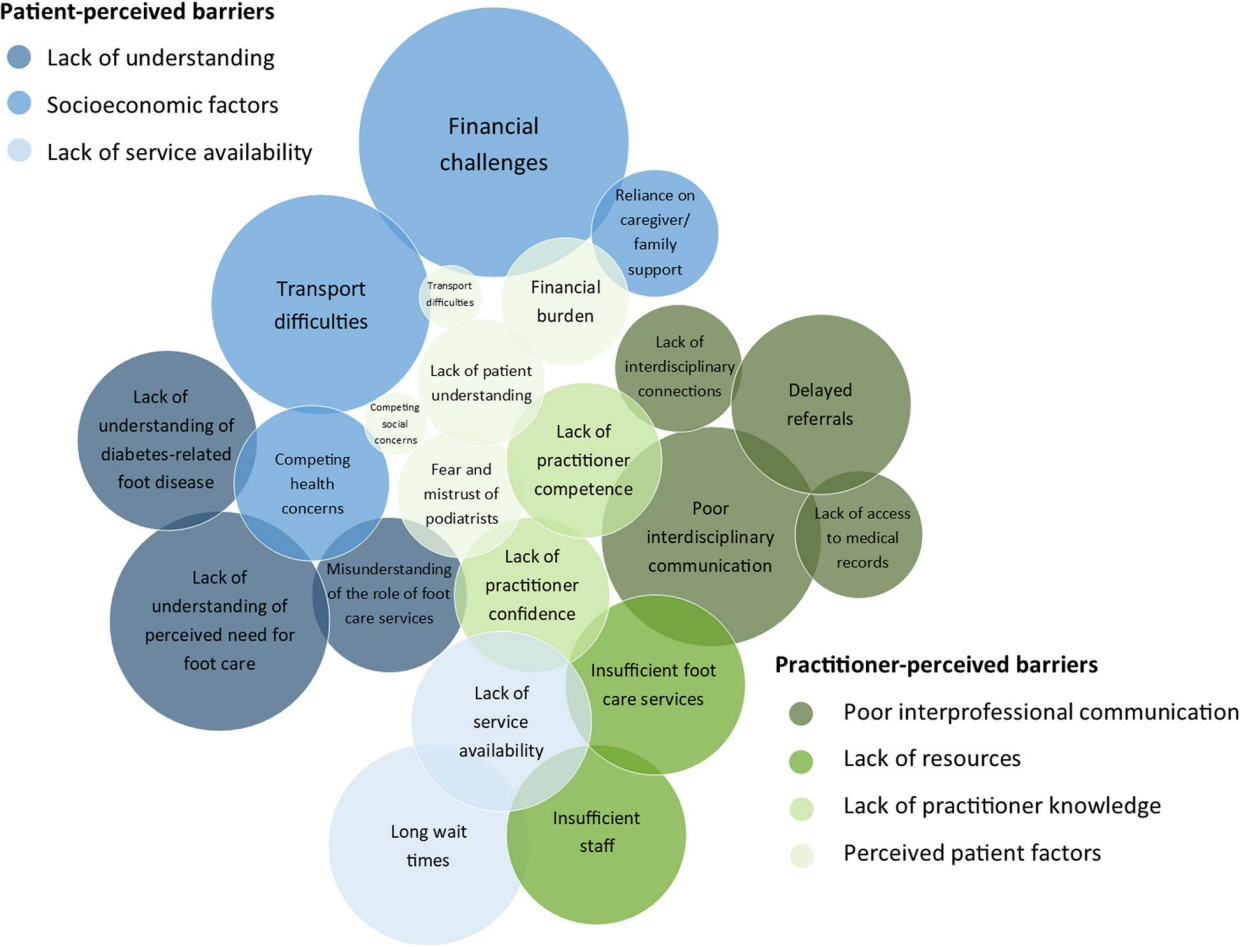
Theme cloud showing subthemes for patient- and practitioner-perceived barriers. The area of the circles represents the number of studies reporting each subtheme

Numerous barriers to accessing diabetes foot care services were acknowledged by both patients and health practitioners. These findings were grouped into themes and subthemes and presented separately for patient-perceived barriers and practitioner-perceived barriers (Fig. 3). The themes identified as patient-perceived barriers were (1) lack of understanding, (2) socioeconomic factors, and (3) lack of service availability. For practitioner-perceived barriers, the predominant themes were (1) poor interprofessional communication, (2) lack of resources, (3) lack of practitioner knowledge, and (4) perceived patient factors.

### Patient-perceived barriers

#### Theme 1: lack of understanding (patient-level factor)

The greatest recurring theme among patient-perceived barriers was a lack of understanding. This theme encompassed three subthemes: (1) lack of understanding of perceived need for foot care, (2) lack of understanding of diabetes-related foot disease, and (3) misunderstanding of the role of foot care services.

Patients with diabetes who were not receiving specialized foot care reported that they were not educated on the potential to develop foot complications by their health care provider or when and how to access foot care services [12, 23, 24, 26]. Some patients felt they did not need regular foot care in the absence of an apparent foot or lower limb problem or if their feet “were not hurting” [12, 26]. Many did not understand how diabetes contributed to their foot injuries or that regular access to foot care services could identify foot complications before they developed [12, 20].

Patients with diabetes also had a general lack of knowledge about diabetes-related foot disease which posed a frequent barrier to accessing foot care services [20, 23, 24]. Patients frequently reported being unable to recognize potential foot problems, and were taken by surprise when a small cut or bruise developed into a significant complication [23, 24]. This resulted in delayed access to foot care services by either attempting at-home care, ignoring symptoms, or believing they would get better in time [19, 20, 23, 24]. One patient stated, “My feet start[ed] going bad...but I didn’t understand, I didn’t know, didn’t no one tell me or I didn’t go to the doctor for that, I just kept on working ...” [19].

Many patients also expressed a misunderstanding of the role of foot care services with some perceiving podiatry as a ‘pedicure’ service [16]. Others did not know specialist foot care services were available to them, until they had been referred after developing more serious foot complications [18]. Rather than seeking care from podiatrists or other foot-specialists, some patients also sought alternative care through chiropractors, homeopaths, or acupuncturists for their foot-related concerns [26].

#### Theme 2: socioeconomic factors (patient-level factor)

In nearly all studies patients described some form of social health determinant impacting their ability to specialist foot care services. Four subthemes emerged from this theme: (1) transport difficulties, (2) financial challenges, (3) reliance on caregivers/family support, and (4) competing health concerns.

Difficulties with transport to and from appointments was a common access barrier, particularly for patients living in rural areas where local foot care services were scarce [17, 23, 26, 30]. Even for patients living in urban areas, there were a number of travel-related barriers, including transport, exhaustion from long rides, severe traffic jams and parking issues [16, 17, 20, 23, 24]. Travel was particularly challenging for patients who required wheelchair accessible transport and parking [17, 20, 24]. Many patients relied on public transport, however not all buses were wheelchair accessible, and claiming reimbursements for private taxi travel was a difficult process [17].

The lack of funding to attend preventative foot care services posed a huge cost barrier to patients [16, 17, 19, 24, 26, 28]. Even with the aid of insurance and medical care cards, patients with low incomes still struggled to pay for foot care services [16, 19, 20], with one patient reporting: “I don’t go to the podiatrist. Every session is fifty-five dollars; I can’t afford it” [16]. Some patients with diabetes-related foot complications were unable to work to earn money [24], while others could not afford to miss work, so missed appointments instead [23, 30].

Reliance on social support to access foot care services was reported among many patients, who required support from family members, spouses, neighbors, or caregivers to attend appointments [17, 24]. Without their support they would not be able to access foot care services.

For many patients, wider social circumstances competed with their health concerns which prevented many from accessing foot care, including housing or changing living arrangements, multiple health issues, and family matters [17, 20, 23]. One patient described that prior to his toe amputation, he missed foot care appointments because, “My…son was going through cancer treatments…I was going back and forth with him to the doctor, and I was focused on that. I wasn’t about to try to take the focus off of that” [23].

#### Theme 3: lack of service availability (system-level factor)

The final theme identified as a patient-perceived barrier was the lack of service and appointment availability which encompassed two subthemes: (1) long wait times, and (2) lack of service availability.

Long wait times were reported at all levels of diabetes foot care including podiatry, primary care, vascular surgery, wound care specialists, ambulatory care, and hospital level care [20, 23, 24]. Living rurally seemed to exacerbate the issue of long wait times, as one patient explained, “I called [an urban specialty clinic] in January. The earliest they could get me in was the first week of April. Really? You’re telling me that she’s booked for the next four months?” [29]. As a result of appointment delays for foot care services, some patients resorted to urgent care facilities, emergency departments of different hospitals [19, 23]. Many patients felt that the long wait times for accessing foot care services contributed to worsening of their foot complications [19].

A complete lack of available foot care services also posed a huge access barrier for patients, with many reporting a great deal of active engagement in order to secure appointments [21, 23, 30]. Due to a lack of foot care services in their area, many patients had to travel to larger hospitals. Some patients had to go to multiple clinics to seek foot care services: “We still needed to go to a big hospital to treat the foot. The community hospital could only change the dressing” [24]. Others that lived rurally reported challenges with availability of mobile foot care services: “When the podiatrist saw me she said, “Who cut you toenails?“ I said, “I do it myself“, and she said, “You shouldn’t do it“, I said “Well I do it myself because you never come to see us“. That’s why I lost my toe. You got to come and see us more often” [30].

### Practitioner-perceived barriers

#### Theme 1: poor interprofessional communication (system-level factor)

Among the studies that examined practitioner perceptions, many reported poor interprofessional communication as a critical barrier for patients accessing foot care services. The four subthemes were: (1) delayed referrals, (2) lack of interdisciplinary connections, (3) poor interdisciplinary communication, and (4) lack of access to medical records.

Delayed referrals, including to specialized diabetes foot clinics or podiatrists, were reported by practitioners as an important access barrier for patients [6, 25]. Delayed referrals were often a result of untimely referral pathways which involved referring back to a general practitioner to refer to the specialist department [6, 29]. Practitioners described the referral pathway to foot care services as complex due to poor understanding of indications for referral, urgency for referral, and whom to refer to [6, 27].

The complexity of standard referral pathways meant that practitioners needed to rely on their own professional links, however, many practitioners lacked the connections required for timely referral of patients to foot care services [22, 29]. “Where do I send someone with a diabetic foot ulcer that’s beyond my skill level?...Time is going by…I ended up admitting him and he got amputated” [29]. Rural health providers also report a lack of connection with urban specialists, with many unable to name a single foot care specialist to refer a patient with a diabetic foot ulcer to [29].

Poor interdisciplinary communication amongst practitioners resulted in difficulties contacting and referring patients to foot care specialists [6, 14, 15, 27]. The lack of joint planning and collaboration, coupled with poor or absent lines of communication among health practitioners, resulted in untimely access to foot care services [27, 29]. With poor or negative communication and unclear roles within the interdisciplinary team, there was not a systematic assessment, triage or collaborative approach to provide patients with smooth access to foot care [22].

Additionally, many practitioners also expressed the difficulty in accessing patient medical records, which also posed a barrier to communication between hospitals and private practitioners [6]. This was reported to impede the ability of practitioners to triage referrals to foot care services and to act on specialists’ recommendations: “We don’t even know if [the primary care provider] saw our note, so we don’t know if they’re considering our recommendation...The recommendation that we made two weeks ago hasn’t happened yet because of the inefficiencies [with electronic health records]” [29].

#### Theme 2: lack of resources (system-level factor)

Lack of resources emerged as another theme described by practitioners as a barrier to patients accessing foot care. The subthemes identified were: (1) insufficient staff, and (2) insufficient foot care services.

Inadequate staff numbers, including podiatrists with specific knowledge and skills in diabetic foot care, was described by many practitioners and was related to an inability to recruit and retain staff, particularly in rural areas [6, 14, 27, 29]. Insufficient funding to appoint practitioners in foot care services was a key factor contributing to staff shortages [6, 27].

Practitioners also commented on the lack of availability of specialty foot care services, including podiatry, vascular teams, multidisciplinary diabetes foot clinics, orthotic services, and orthopedic clinics [6]. Rural communities experienced the greatest lack of foot care service availability across a number of studies [6, 14, 18], with many rural settings providing no clinical podiatry care to people with diabetes [14]: “There isn’t a high-risk foot service. The closest is a plane ride or a six-hour drive” [27]. Insufficient funding and investment in preventative foot care services and resources/equipment required to deliver them played a key role in service availability for people with diabetes [6, 27]. Some practitioners also reported witnessing postponed foot care appointments for their patients if another patient had a more serious condition [27].

#### Theme 3: lack of practitioner knowledge about foot care (system-level factor)

Practitioners frequently reported a lack of knowledge about diabetes-related footcare, which was identified as another major theme and barrier to accessing foot care services, encompassing two subthemes: (1) lack of practitioner competence, and (2) lack of practitioner confidence.

Many practitioners felt they did not receive sufficient education on providing diabetes foot care [6, 27]. This lack of perceived competence meant some practitioners were resistant to taking on new patients with a foot care focus [21]. Although nurses and general practitioners were viewed as having a lack of skills and knowledge about foot care (“often, you’re dealing with GPs who have no idea about high-risk feet”) [27], so too were podiatrists: “Not all podiatrists know about all the diabetes footcare” [27].

A lack of knowledge about diabetic-specific foot care directly translated into a lack of confidence, including a lack of their own confidence, as well as a lack of confidence in other health professionals, including podiatrists [6, 14, 27]. Practitioners who had low confidence in managing diabetes related foot complications were reluctant to provide foot care services [27]. Reasons for mistrust in other practitioners was due to inaccurate diagnoses, having poor awareness of the urgency required for a referral to specialist care, and a lack of education about foot inspections, screening, and referrals [6].

#### Theme 4: perceived patient factors (patient-level factor)

The final theme arising from practitioner perspectives was perceived patient factors, which paralleled many of the patient-perceived factors identified above. Subthemes included: (1) lack of patient understanding, (2) fear and mistrust of podiatrists, (3) financial burden, (4) transport difficulties, and (5) competing social concerns.

Practitioners recognized that patients lacked understanding about diabetes foot complications and the importance of access to regular foot care services; only accessing them once a serious problem had occurred [6, 21].

Some podiatrists also felt there was a sense of fear and mistrust among patients who had more trust for the doctors: “[podiatrists are only] here to cut nails” [21]. Other health practitioners described that patients’ feared podiatrists and would avoid podiatry appointments [13].

Some practitioners also perceived their patients’ financial situations posed a barrier to specialist foot care service access, with many patients showing up to public systems to avoid private health care costs [21, 27].

Limited transport options were also acknowledged by practitioners as a patient-level access barrier, especially when escalation of care was needed, and travel to appointments was costly and time-consuming [27].

Health practitioners were also aware of wider social factors that acted as a barrier to their patients accessing foot care services: “other issues get in their way, the struggle with poverty, finding a job, the expense of better foods” [21].

## Discussion

This review identified barriers to accessing diabetes foot care services from both the patient and practitioner perspective. Despite clear overlap between the two perspectives, most patient-level factors (i.e., socioeconomic factors and lack of understanding) were identified by the patients themselves, with little acknowledgment from the practitioners, who focused primarily on barriers related to the health-care system (i.e., poor interprofessional communication).

The most commonly reported patient-perceived barrier to accessing footcare services was associated with the financial challenges of paying for and travelling to appointments. This is consistent with many aspects of diabetes management, including self-management, obtaining medications, and affording healthy food [31]. Although financial difficulties are associated with reduced physical and mental wellbeing for people with diabetes [32], practitioners are often insensitive to these financial barriers [31]. This was also reflected in the current review, in which financial difficulty was the least commonly recognized access barrier from the practitioner perspective and highlights the need for ongoing practitioner training in “financial sensitivity” [32].

A lack of understanding of diabetes related foot disease and the perceived need for foot care was a common barrier identified by patients that prevented them from accessing foot care services. It has been well-established that patients with diabetes lack knowledge about foot complications and their prevention [33–36]. A lack of knowledge about one’s condition and how it is managed has been shown to cause a low perceived need and reduction in care seeking across many chronic conditions [7]. Improving patient engagement through health education and availability of information can be achieved through innovative technology (i.e., apps, telemedicine, and social media) that promote patient empowerment and knowledge about their condition. Improving patient engagement in foot care services through the use of technology has been shown to successfully reduce amputation rates in people with diabetes [37].

Although many global frameworks exist for effective interdisciplinary management of the patient with diabetes, they are not widely understood or utilized [38]. In the current review, the most commonly reported access barrier from the practitioner perspective was related to poor interdisciplinary communication. Clear and open communication within the team is among the many factors contributing to successful interdisciplinary management, and includes collaborative and supportive relationships, shared goals, and diversity of expertise [39]. Without functioning teams, appropriate and timely referral pathways to foot care services are lost, leading to worse patient outcomes.

The findings from this review should be considered in light of some limitations. Firstly, the findings may not apply to all global populations, as studies from only ten countries met the criteria for inclusion, the majority of which were from high-income countries. The barriers identified in this study may not be applicable to other regions where cultural and ethnic differences may impact service access. Secondly, all but one study in this review took place prior to the Covid-19 pandemic, and it is likely the results from this review do not reflect access barriers related to Covid-19 (including lockdowns, high health care demand, and staff shortages) [40]. Finally, although the methodology was designed to increase study rigor and reduce bias through the use of multiple authors when coding and thematizing data, it cannot be ruled out that unintentional subjectivity may have influenced the analysis.

## Conclusions

In conclusion, the findings from this study have demonstrated a number of barriers to accessing foot care services from both the patient and practitioner perspectives. Although patients focused predominantly on patient-level factors, while practitioners focused on barriers related to the health care system, there was some overlap between them. This emphasizes the importance of recognising both perspectives for the future integration of policy changes and implementation of access facilitators.

## Data Availability

The datasets used and/or analysed during the current study are available from the corresponding author on reasonable request.

## Abbreviations

CASP: Critical Appraisal Skills Program
NHLBI: National Heart, Lung & Blood Institute
PRISMA: Preferred Reporting Items for Systematic Reviews and Meta-Analyses
NR: Not reported
NA: Not applicable
